# Geographical variability of bacterial communities of cryoconite holes of Andean glaciers

**DOI:** 10.1038/s41598-022-24373-5

**Published:** 2023-02-14

**Authors:** F. Pittino, R. Ambrosini, M. Seeger, R. S. Azzoni, G. Diolaiuti, P. Alviz Gazitua, A. Franzetti

**Affiliations:** 1 Department of Earth and Environmental Sciences (DISAT), Università degli Studi di Milano-Bicocca, Milan, Italy; 2 WSL Swiss Federal Research Institute, Birmensdorf, Switzerland; 3 Laboratory of Glacier Ecology, Department of Environmental Science and Policy, Università degli Studi di Milano, Milan, Italy; 4 Molecular Microbiology and Environmental Biotechnology Laboratory, Department of Chemistry, Universidad Técnica Federico Santa María, Valparaiso, Chile; 5 Department of Earth Science “Ardito Desio”, Università degli Studi di Milano, Milan, Italy; 6 Departamento de Ciencias Biológicas, Universidad de los Lagos, Osorno, Chile

**Keywords:** Microbial ecology, Microbial communities

## Abstract

Cryoconite holes, ponds full of melting water with sediment on the bottom, are hotspots of biodiversity on glacier surfaces and host dynamic micro-ecosystems. They have been extensively investigated in different areas of the world (e.g., the Arctic, Antarctic, Alps, and Himalaya), but so far no study has described the bacterial communities of the glaciers in the Andes, the world’s longest mountain range. In this study, we describe the bacterial communities of three small (< 2 km^2^) high-elevation (< 4200 m a.s.l.) glaciers of the Central Andes (Iver, East Iver and Morado glaciers) and two large (> 85 km^2^) glaciers of the Patagonian Andes (Exploradores and Perito Moreno glaciers) whose ablation tongues reach low altitude (< 300 m a.s.l.). Results show that the bacterial communities were generally similar to those observed in the cryoconite holes of other continents, but with few cyanobacteria (0.5% of sequences). The most abundant orders were Betaproteobacteriales, Cytophagales, Chitinophagales, Acetobacterales, Frankiales, Armatimonadales, Sphingobacteriales, Rhizobiales, Bacteroidales, Sphingomonadales, and Micrococcales. The bacterial communities differed between glaciers and both water pH and O_2_ concentration appeared to influence the bacterial community composition. This work thus provides the first description of the bacterial communities in cryoconite holes of South American glaciers.

## Introduction

Glaciers and ice sheets have been recognized as a biome in their own right, mostly dominated by microorganisms that live in all parts of the glacier environment^1^. The supraglacial zone is the most biodiverse among glacier ecosystems^1^ and, for practical reasons, is also the most studied. Here, small holes of the glacier surface filled by meltwater, the cryoconite holes, form due to the atmospheric deposition of fine-grained sediment, the cryoconite, which locally decreases the albedo and melts the underlying ice. The main source of this sediment is the surrounding environment, but a small part of it is deposited after long-range atmospheric transport^2^. In the extreme glacier environment, cryoconite holes are protected habitats where general conditions are more favourable for life than in the other parts of a glacier, as they provide liquid water which also acts as a protective barrier against the intense UV radiation characteristic for glacier environments^3^.

Cryoconite holes are present on glaciers in almost all areas of the world; so far they have been described with their microbiome in the Arctic, Antarctica, the Alps, Tien Shan, Karakoram, Himalaya and Caucasus^2,4–8^. However, they do not show the same features in all the different geographic areas where they occur. For instance, on the Greenland Ice Sheet and in Antarctic regions, cryoconite holes are more stable than those that form on temperate mountain glaciers. On Himalayan glaciers, they can sometimes last for more than one year^7,9^, but on other temperate mountain glaciers as in the Alps, they are mostly ephemeral structures that can be destroyed by strong ablation that can quickly dismantle them washing away the sediment. The intense solar radiation can subsequently form new holes in a few days^10^.

Investigations of the temporal variability of cryoconite hole bacterial communities of the same glacier along one or even more ablation seasons were seldom conducted^10,11^, mostly because of the difficulties and the costs of visiting glaciers repeatedly, as they usually occur in remote areas. Most often, the description of the bacterial communities of cryoconite holes is conducted with snapshot studies^12^, which allow a general description of their biotic communities, even if they cannot include the whole biodiversity of these environments^10,13^. These studies showed that filamentous cyanobacteria are the main organisms responsible for cryoconite grain formation^7,8^. Other abundant bacterial phyla found in these microhabitats are proteobacteria (alpha and beta), actinobacteria, chloroflexi, acidobacteria and bacteroidetes^5,14^. Cryoconite hole bacterial communities change among glaciers^15,16^ probably according to several different environmental variables (e.g. lithology of the surrounding mountains, elevation, geographic location), as well as within glaciers among cryoconite holes^4,17–19^. Within-glacier variation was related to micro-environmental conditions of the single holes. For instance, on Forni Glacier (Italian Alps) water pH and oxygen concentration, the amount of organic matter in the sediment, and the depth of cryoconite holes were related to the bacterial community structure^13^. On Baltoro Glacier (Pakistani Karakoram) the structure of bacterial communities appeared to vary according to the water pH of cryoconite holes^4^. Other variables related to within-glacier variation in cryoconite holes bacterial communities were temperature, solar radiation, wind exposition, electrical conductivity, ammonium, and phosphorous^10,15,20,21^.

To the best of our knowledge, no published information is currently available about cryoconite hole bacterial communities from South American glaciers. Only two studies described cryoconite characteristics of South American glaciers, but from a physico-chemical point of view, not reporting bacterial communities composition^9,22^. Another study describing the gut microbiome of the glacier stonefly (*Andiperla willinki*) with 16S rRNA sequencing also reported some data about the composition of the bacterial communities of the glacier surface because these insects likely feed on supraglacial bacteria^23^, but no study specifically focused on the bacteria inhabiting cryoconite holes on glaciers in South America. This is a big gap of knowledge since the Andes host many glaciers (~ 32,558 km^2^ of glacierized areas in Chilean and Argentinian Andes alone) along a latitudinal range of ~ 8000 km from 11° N in Colombia and Venezuela to 55° S in Chile and Argentina^24,25^.

**Figure 1:**
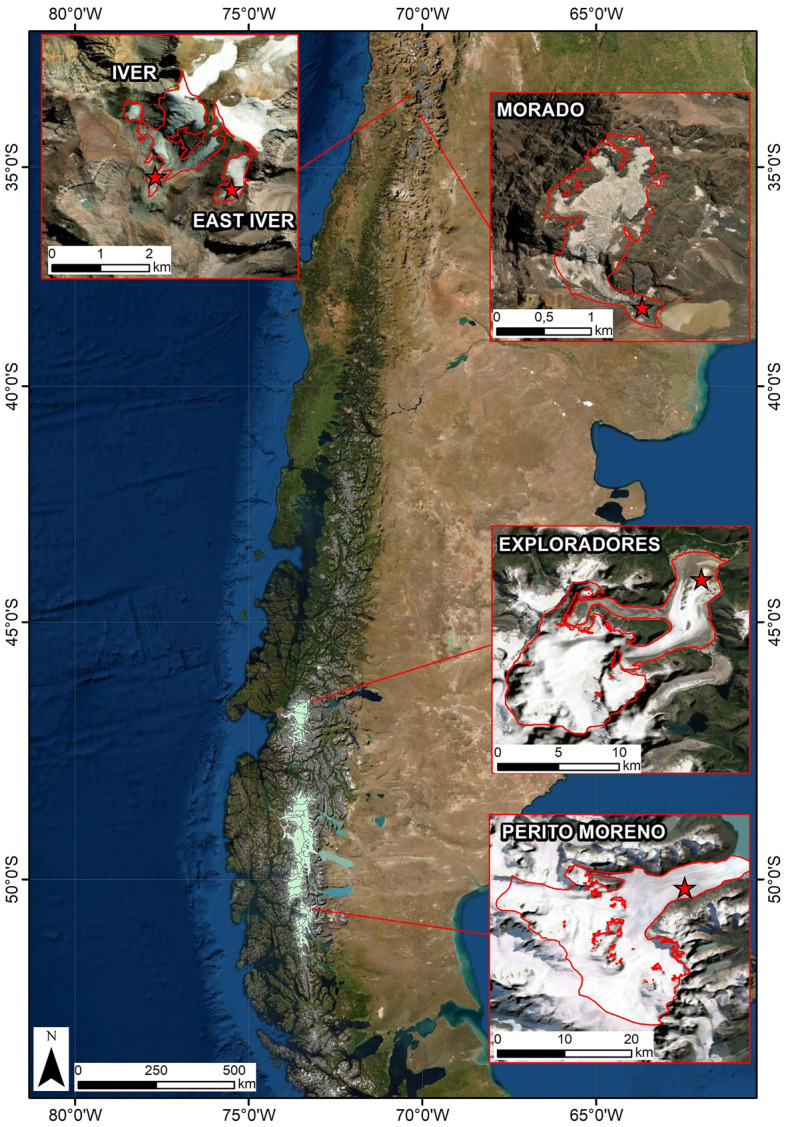
Location Map of Andean glaciers located in Chile and Argentina analysed in this report (ArcGIS Online map hosted by Esri). Glacier limits and morphological parameters were obtained from the Randolph Glacier Inventory 6.0 (RGI Consortium 2017). Stars indicate the position of the sampling areas.

This work aims to compare the bacterial communities from one Argentinian and four Chilean glaciers differing in their geographical and ecological settings. Three of them (Morado, Iver and East Iver glaciers) are small (< 2 km^2^) glaciers located at high altitudes (about 4000 m a.s.l.) in the Central Andes close to Santiago (Chile), while two (Exploradores and Perito Moreno) are large (> 8 km^2^) glaciers located respectively in Chilean and Argentinian Patagonia whose ablation zones (where we collected samples) reach low altitudes (about 300 m a.s.l.; Fig. 1). The locations of these glaciers will shed light on the geographical variability of the bacterial communities in different parts of the Andes as well as on their relationships with two basic parameters of the cryoconite hole environment, particularly pH and oxygen concentration^4,10,13^. Overall, this work provides the first description of the bacterial communities of cryoconite holes of South American glaciers, a geographical area where the cryosphere is rapidly changing due to global warming^25^. The massive reduction in glacier extent and mass that is occurring worldwide is indeed considered a major threat to the understudied glacier biodiversity that is at high risk of disappearing even before being described^26,27^.

## Results

**Figure 2:**
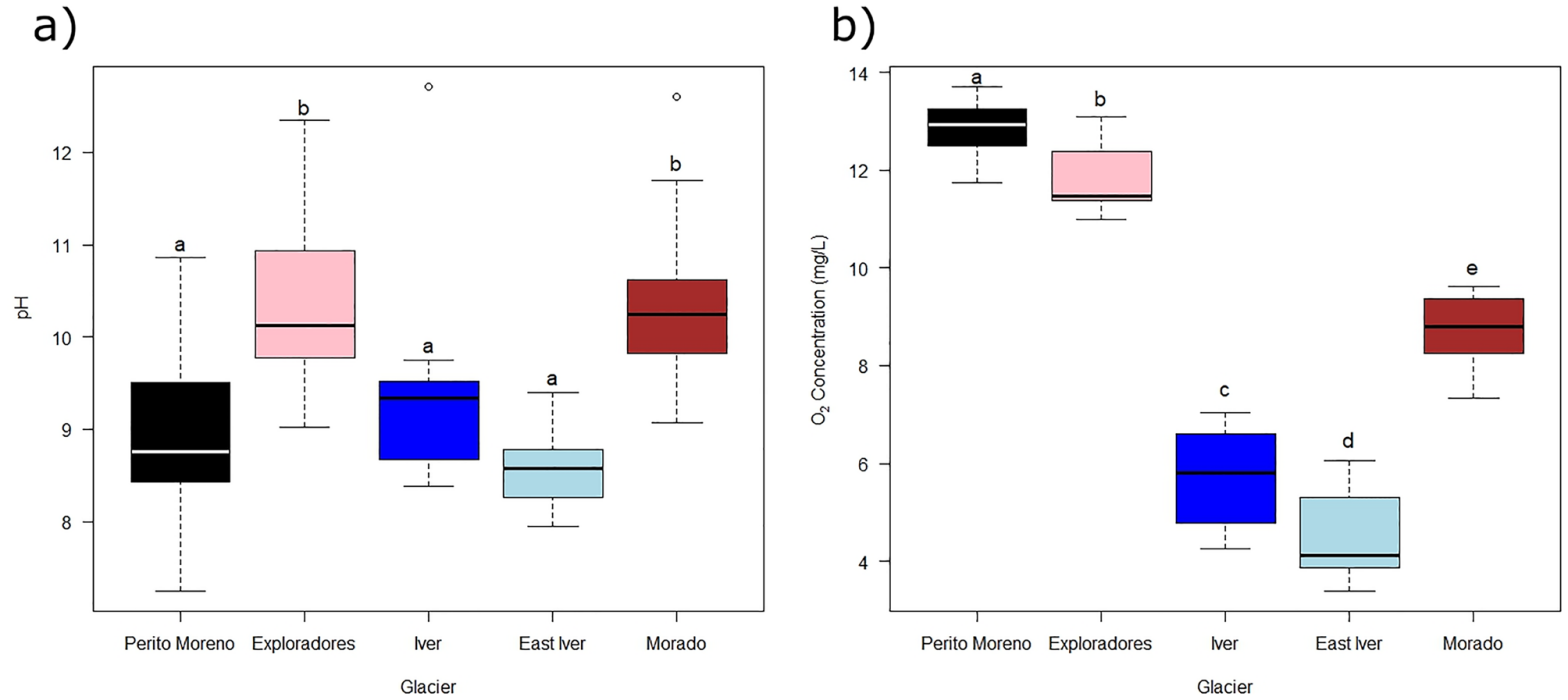
Boxplots of pH (**a**) and oxygen concentration (**b**) of cryoconite holes in the investigated South American glaciers. The thick lines represent the median, boxes upper and lower limits the 25th and the 75th percentiles respectively, whiskers the 5th and the 95th percentiles respectively, dots represent the outliers and different letters indicate significant differences between glaciers at post-hoc tests.

ANOVA tests showed that both pH (F_4,70_ = 11.87, P < 0.001) and oxygen concentration (F_4,70_ = 281.9, P < 0.01) in cryoconite holes differed significantly between glaciers. Tukey post-hoc tests highlighted that glaciers could be divided into two groups with respect to pH values, with Exploradores and Morado showing higher values than Perito Moreno, Iver, and East Iver (|t_70_| ≥ 0.147, P ≤ 0.035; Fig. 2a), while all glaciers differed to one another in their oxygen concentrations (|t_70_| ≥ 3.480, P ≤ 0.011; Fig. 2b).

**Figure 3:**
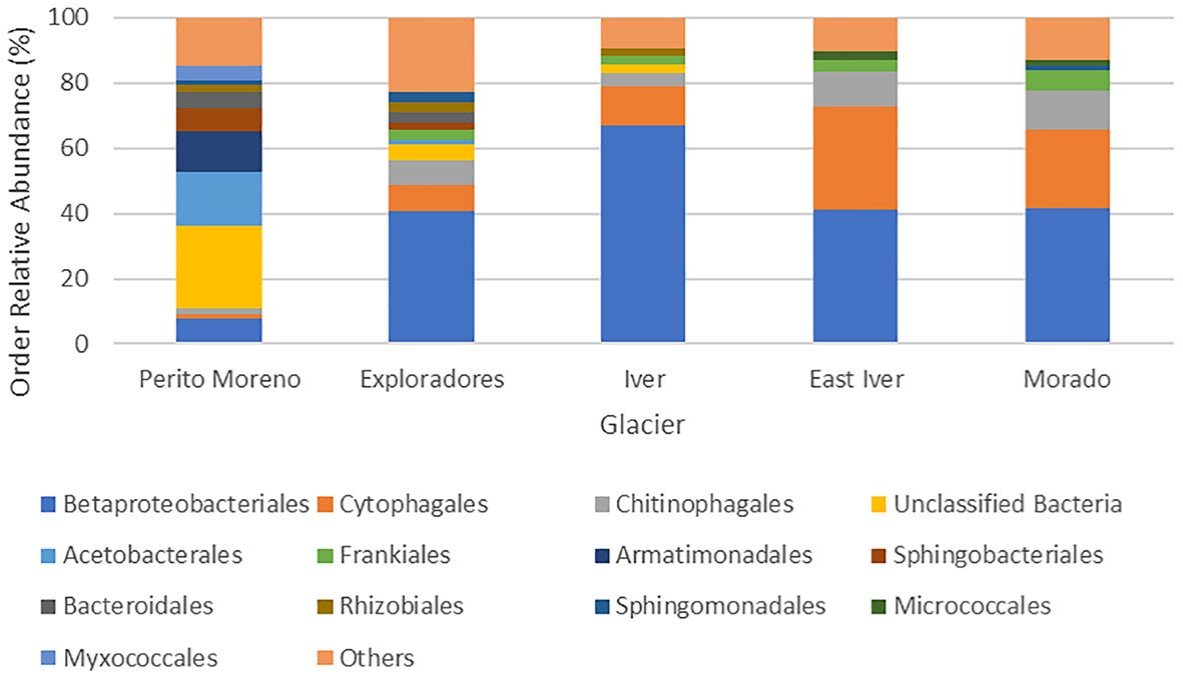
Barplot showing the relative abundance of the most abundant bacterial orders in the cryoconite holes on each glacier.

Using Illumina sequencing, we obtained 7600–93,407 sequences of the V5–V6 hypervariable region of the 16S rRNA gene per sample. Six samples from Morado Glacier had a very low number of sequences (241–816) per sample and were discarded from the analyses of the alpha diversity. However, they were included in the analyses of beta diversity because they clustered with the other samples, and the results did not change when we re-ran the analyses excluding them (details not shown). Overall, these sequences clustered in 1467 Amplicon Sequences Variants (ASVs)^28^. Orders with more than 24,000 sequences were considered the most abundant ones. They were: Betaproteobacteriales, Cytophagales, Chitinophagales, Acetobacterales, Frankiales, Armatimonadales, Sphingobacteriales, Rhizobiales, Bacteroidales, Sphingomonadales, and Micrococcales. Together these orders include 79.8% of sequences (Fig. 3). Overall, the phylum cyanobacteria accounted for only 0.5% of sequences.

Generalized least squares (GLS) models showed that alpha diversity indices varied significantly between glaciers (Shannon index: F_4,62_ = 42.56, P < 0.001; Gini index: F_4,62_ = 32.63, P < 0.001). Post-hoc tests showed that the Shannon index was higher in Exploradores samples, intermediate in Perito Moreno and Morado ones, and lower in Iver and East Iver ones, while the Gini index was higher in Iver and East Iver samples, intermediate in Perito Moreno and Morado ones, and lower in Exploradores ones (Fig. S1). In the same GLS models, we related these indices to pH and oxygen concentration ([O_2_]). However, since the mean values of pH and [O_2_] differed between glaciers (see above), we mean-centred these variables within glacier before the analyses (i.e. we subtracted from the recorded values their mean value within-glacier) thus obtaining ΔpH and Δ[O_2_] values for each sample. This procedure was necessary because mean-centred variables were not collinear with the glacier factor. According to this analysis, no trends were observed according to ΔpH (F_1,62_ ≤ 0.449, P_FDR_ > 0.5) and Δ[O_2_] (F_1,62_ ≤ 0.449, P_FDR_ > 0.5).

**Figure 4:**
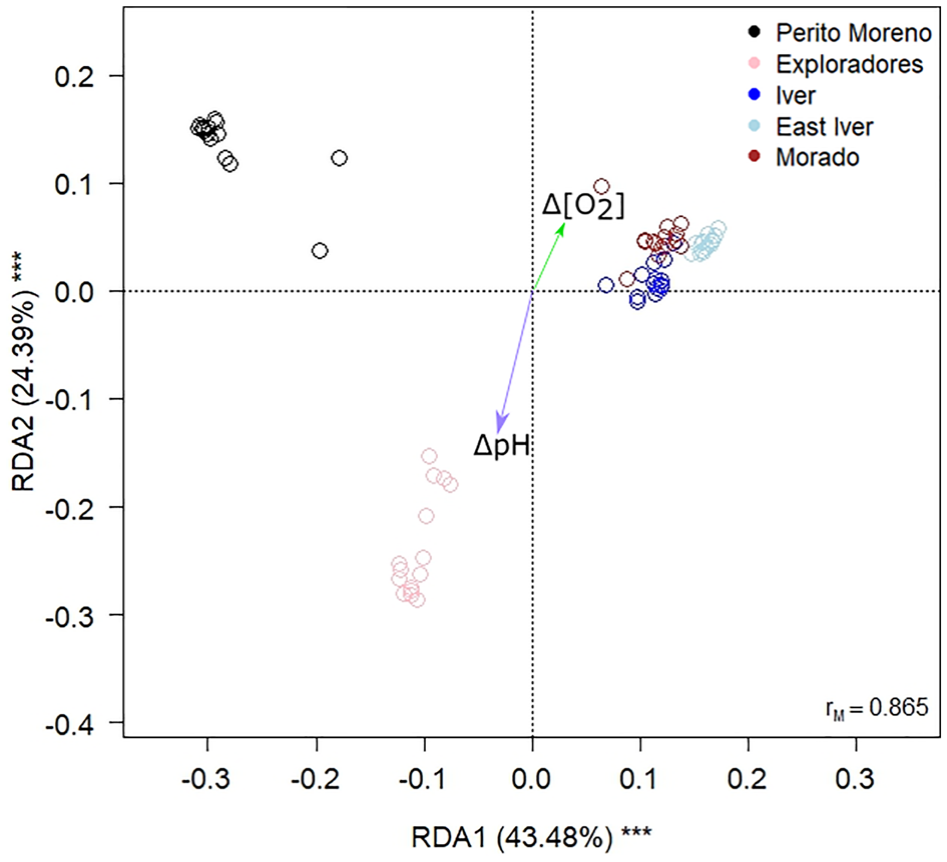
Correlation biplot from RDA on Hellinger-transformed bacterial ASVs abundances of all the samples according to glacier (five-level factor), pH and oxygen concentration centred to their mean value per glacier. The green arrow indicates increasing values of oxygen concentration in each glacier, while the violet one increasing pH values in each glacier. The percentage of variance explained by each axis and their significance (***:P < 0.001) is reported. r_M_ is the Mantel correlation coefficient between the Hellinger distances between samples and the Euclidean distances between the corresponding symbols in the graph. Values close to one indicate that the graph correctly represents the distance between samples.

We used redundancy analysis (RDA) to investigate variation in bacterial communities between glaciers and with ΔpH and Δ[O_2_]. This analysis showed that the bacterial communities differed significantly among glaciers and varied according to ΔpH and Δ[O_2_] (Table 1; Fig. 4). The biplot also suggested that cryoconite hole bacterial communities of the three small glaciers in Chilean Central Andes (Iver, East Iver and Morado) varied mostly according to Δ[O_2_], while those of the Exploradores glacier in Patagonian Andes vary according to ΔpH. Post-hoc tests also revealed that the structures of bacterial communities of all five glaciers were significantly different from one another (F_1,68_ ≥ 21.527, P_FDR_ ≤ 0.001).

**Table 1:** RDA of Hellinger-transformed bacterial ASVs abundances of all the samples according to the glacier, pH and oxygen concentration centred according to their mean value per glacier.

Variable	Df	Variance	F	P
Glacier	4	0.432	39.61	0.001
ΔpH	1	0.005	1.98	0.015
Δ[O_2_]	1	0.006	2.13	0.001
Residuals	68	0.181		
Overall model: F_6,68_ = 27.056, P = 0.001, Adjusted R^2^ = 0.679

**Figure 5:**
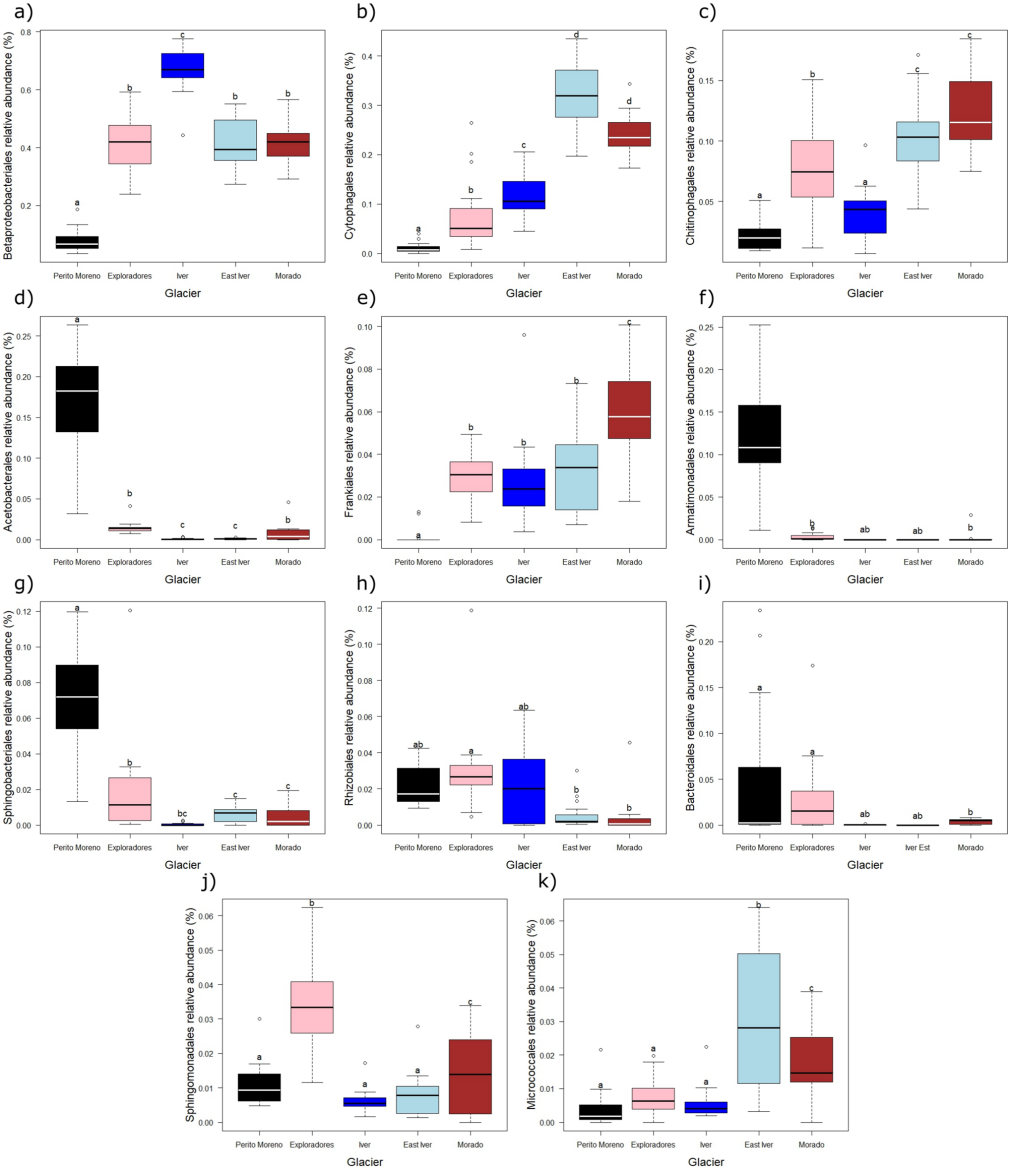
Boxplots of the relative abundances of Betaproteobacteriales (**a**), Cytophagales (**b**), Chitinophagales (**c**), Acetobacterales (**d**), Frankiales (**e**), Armatimonadales (**f**), Sphingobacteriales (**g**), Rhizobiales (**h**), Bacteroidales(**i**), Sphingomonadales (**j**) and Micrococcales (**k**) on the five glaciers where we collected cryoconite samples. The thick lines represent the median, boxes upper and lower limits the 25th and the 75th percentiles respectively, whiskers the 5th and the 95th percentiles respectively, dots represent the outliers and different letters indicate differences between the mean values of different groups.

Poisson generalized linear models (GLMs) corrected for overdispersion performed on the most abundant orders showed that the abundances of Betaproteobacteriales, Cytophagales, Chitinophagales, Acetobacterales, Frankiales, Armatimonadales, Sphingobacteriales, Rhizobiales, Bacteroidales, Sphingomonadales, and Micrococcales varied between glaciers (F_4,68_ ≥ 6.181, P_FDR_ < 0.001), without showing any consistent pattern. Iver was the glacier with the highest relative abundance of Betaproteobacteriales, Morado the one with the highest relative abundance of Frankiales, East Iver showed the highest abundance of Cytophagales and Micrococcales, and Perito Moreno had more Armatimonadales, Sphingobacteriales, and Acetobacterales, but fewer Betaproteobacteriales, Frankiales and Cytophagales than the other glaciers (Fig. 5).

The five glaciers we considered showed different pH and [O_2_] values, and the RDA biplot suggested that the communities of different glaciers were affected by different environmental variables. We therefore investigated the relations between each of the most abundant orders and pH and O_2_ concentration on each glacier separately. Similarly, we analysed the relations between alpha diversity indices, pH, and [O_2_] separately per glacier. Only on the Exploradores Glacier, Bacteroidales increased with pH (F_1,12_ = 14.275, P_FDR_ = 0.0335) (Fig. S2). Results did not change when we re-run the analyses excluding the six Morado samples with few sequences (details not shown). Shannon and Gini indices did not show any trend according to pH (F_1,12_ ≤ 1.244, P ≥ 0.307) or [O_2_] (F_1,12_ ≤ 1.970, P ≥ 0.186).

## Discussion

In this study, we provide the first description of the bacterial communities of cryoconite holes from South American glaciers, in particular from both small high-elevation glaciers of the Central Andes in the Santiago Metropolitan Region (Chile), and from the tongues of two large glaciers in Patagonian Andes that reach low altitudes. These pieces of information fill a large geographical gap in our knowledge of glacier environments because this is the first description of the microbial communities of supraglacial environments in South America, a continent with about 30,000 km^2^ covered by ice^29^. Results showed that the large Patagonian glaciers (Exploradores and Perito Moreno) had the highest oxygen concentrations, while Iver and East Iver had the lowest ones and Morado an intermediate value. This pattern could be related to the different altitudes of the glaciers. Indeed, since water temperature in cryoconite holes is always quite low and stable at all altitudes, oxygen solubility in these environments is related to the atmospheric partial pressure of oxygen that decreases at increasing altitude^30^. This result is consistent with [O_2_] values we found in our samples. Indeed, Exploradores and Perito Moreno are located in Patagonia at low altitudes (< 200 m a.s.l.), while Iver and East Iver are the highest ones among those we investigated (samples were collected at about 4000 m a.s.l.) and Morado is at an intermediate value (3400 m a.s.l.).

pH values seem to follow a different pattern: the highest values were recorded on Exploradores and Morado, while the lowest ones were on East Iver and Iver. However, all the mean pH values of the five glaciers were basic (between 8.57 and 10.47) and differences in pH among glaciers are not easily explained also considering the lithology of the surrounding environments. Indeed, Perito Moreno lies between rhyolitic and undifferentiated volcanic bedrock, Morado, Iver and East Iver are located between alkaline and andesitic igneous rocks, and Exploradores is characterized by a granitic bedrock^31^. We note, however, that we measured water pH rather than that of the cryoconite because the first one was already reported to influence bacterial communities in cryoconite holes^4^.

The most abundant orders were (in decreasing order): Betaproteobacteriales, Cytophagales, Chitinophagales, Acetobacterales, Frankiales, Armatimonadales, Sphingobacteriales, Rhizobiales, Bacteroidales, Sphingomonadales, and Micrococcales*.* These orders belong to the phyla Proteobacteria, Actinobacteria, Bacteroidetes, and Armatimonadota. Most of these orders are typical of cryoconite holes and dominate bacterial communities in these environments in all the geographical areas investigated so far: Arctic^32^, Antarctica^5^, Europe^10,12^, and Asia^33^. Betaproteobacteriales are a quite heterogeneous order where diverse members showed resistance to stressful conditions including oxidative stress and also degradation of aromatic compounds^34^. Interestingly, 48.12% of Betaproteobacteriales sequences in our samples belong to the genus *Polaromonas*, a bacterium well adapted to glacier environments^35,36^. According to our results, *Polaromonas* was quite abundant on all the glaciers but on Perito Moreno. This is an important genus because it is well adapted to harsh environments because of its ability to survive long-range atmospheric transport and thanks to its versatile metabolism (can use both organic and inorganic electron donors)^35^. Cytophagales are Gram-negative bacteria that are known to be less resistant to UV radiation than Gram-positive bacteria^37^. In addition, 91.68% of Cytophagales sequences belong to *Hymenobacter*, a heterotrophic genus composed of mostly heterotrophic, aerobic, Gram-negative bacteria which have been described in extreme environments like Antarctica, where a strain with extremely high resistance to solar radiation was isolated^38,39^. Armatimonadota and Frankiales were never reported as abundant inhabitants of cryoconite holes in other geographical areas. Interestingly, Armatimonadota were present with a high relative abundance on Perito Moreno only. This phylum includes both aquatic and terrestrial genera, surprisingly described in hot springs and geothermal soils^40^. Maybe, the presence of pigments, oligotrophy and the ability to degrade polysaccharides provide them with the ability to survive in such harsh environments^40^. Frankiales, together with Micrococcales, belong to the phylum of Actinobacteria and are Gram positive bacteria^41^. They were abundant on all glaciers, except for Perito Moreno and their high relative abundance may be related to their resistance to UV radiation^42^. cyanobacteria, which are considered important components of cryoconite hole bacterial communities and even ecosystem engineers in these environments^9^, were only 0.5% in our samples and no order was included among the most abundant ones. In summary, cryoconite holes of Andean glaciers seem to host bacterial communities dominated by the taxa typical of these environments worldwide, but with a low abundance of cyanobacteria, and a rather high abundance of Frankiales in all glaciers except for Perito Moreno, where a high abundance of Armatimonadota, another unusual taxon in cryoconite holes, was observed.

Despite this general similarity of the dominant orders of bacterial communities at a global scale, a closer inspection at the ASV level revealed that the bacterial communities of the different glaciers differed from one another. In addition, the RDA biplot showed that the three small and high-elevation glaciers clustered quite close to one another, whereas Perito Moreno and Exploradores were separate from one another and the other glaciers. This pattern may be due to geographic distance, different altitudes, or other ecological and environmental differences among glaciers. Indeed, bacterial communities in high-elevation glaciers are exposed to higher UV radiation and, therefore, to high oxidative stress^3^ as well as lower partial oxygen pressure and lower nutrient availability from aeolian deposition. Patagonian glaciers are 440 km from one another and c.ca 2000 km from the other glaciers we sampled, which, in turn, are less than 60 km from one another. In addition, it has been demonstrated that the main source of cryoconite bacterial communities is the local environment surrounding glacier^2^. The high-elevation glaciers we sampled are small and above the tree line, and generally in areas with sparse vegetation. In contrast, the Patagonian glaciers are large and their tongues (where we collected the samples) are well below the tree line and therefore exposed to very different potential sources of microorganisms with respect to the other glaciers.

The RDA also showed that bacterial communities of cryoconite holes varied significantly with ΔpH and Δ[O_2_]. We stress that these variables represent the difference between the pH and oxygen concentration of each cryoconite hole from the respective mean value of all the holes of that glacier. They therefore do not account per se for the difference in mean pH and oxygen concentration among glaciers highlighted in the analyses discussed above, because in this analysis this effect was accounted for by the glacier factor, which accounts for any difference between glaciers. Thus, this analysis suggests that bacterial communities vary consistently according to pH and oxygen concentration gradients present on each glacier, even if different glaciers show on average different pH and oxygen concentration values. In other words, for example, an increase of two pH units seems related to a similar variation in bacterial communities independently from the absolute pH value, at least in the range of variability of pH values recorded in this study. In addition, no effect of ΔpH on alpha diversity resulted from GLSs. pH is known to affect bacterial communities of cryoconite holes^4^, but little is known about the mechanisms underlying these relationships. Indeed, water pH may also be partially affected by the metabolic activities of the bacterial communities, so it is still unclear whether water pH affects cryoconite hole bacterial communities or, at least, partially, also the reverse occurs. Indeed, it has been reported that the photosynthetic activity of both bacteria and algae can alter CO_2_ concentration which, in turn, can alter pH^43^. In addition, in cryoconite holes, this activity can also be balanced by that of the heterotrophic bacterial community and other heterotrophic taxa (e.g. tardigrades, rotifers, fungi)^44^. Furthermore, analyses of the most abundant orders within each glacier revealed that some varied according to pH. Different studies already investigated the effect of both water and sediment pH on bacterial community composition, proving that they influence the communities of different types of river sediments^45^. Nonetheless, so far only the decrease of Acidobacteria at basic pH values was already reported in literature data^45^ but was not detected in our study, probably because the pH values of our samples varied from 7.25 to 12.71 i.e. in a range where the abundance of Acidobateria is always low^46^.

Cryoconite holes are aerobic environments in the water, thanks to the high O_2_ solubility in cold environments, the release of air bubbles from the ice that melts because of the presence of the dark sediment, and to photosynthesis^47^. On these glaciers, we observed on average lower oxygen concentrations in the high-altitude cryoconite holes probably because water oxygen depends on the equilibrium with the atmospheric oxygen. In addition, variation in oxygen concentration in the water seems to play a role in explaining the structure of bacterial community structures, albeit none of the most abundant orders seemed to vary significantly according to [O_2_] concentrations within a glacier. Interestingly, no differences in cyanobacteria abundance were observed, indeed it would be expected that their abundance was higher when also [O_2_] was higher^13^, because of their photoautotrophic activity, but no evidence emerged about it. In any case, the mechanisms that link oxygen concentration in the water to bacterial community structure in the sediment are not easy to explain. Indeed, [O_2_] in the sediment can largely differ from that in the water, with the rapid formation of anoxic layers under a few millimetres of cryoconite^48,49^.

The Iver and East Iver glaciers are the two geographically closest glaciers (only 1.2 km apart), and they show also similar pH and [O_2_] values (Fig. 2a, b) and similar alpha diversity values. In addition, their bacterial communities, although significantly different, were close to one another in the RDA biplot. Interestingly, also the Morado glacier clustered close to them in the RDA biplot, even though it is about 60 km from them. In contrast, the two Patagonian glaciers are separated from one another and the other glaciers on the same plot. These results, on the one side, support the hypothesis that altitude and geographic position play an important role in defining bacterial community composition. On the other side, however, the three small glaciers in the central Andes are also at a similar elevation and they are surrounded by very similar environments (R. Ambrosini and F. Pittino, personal observation). These three high mountain glaciers have also an important anthropic input of aerosol and black carbon that act as sources of cryoconite. Indeed, a high percentage of black carbon deposition on ice in the Central Andes close to Santiago has been associated with emissions from the city (> 40%), whereas mining is also an additional important black carbon source^50^. Their similarity can therefore derive also from being exposed to the same general ecological conditions, including high UV radiation, oxidative stress, anthropic pressures, and probably, also from similar sources of bacteria. These results therefore highlight that correlative studies like the present ones can hardly disentangle the effects of geographical positions and ecological conditions on the structure of cryoconite hole bacterial communities, and further studies should be designed to add insight into this still open question.

Analyses of alpha diversity indices indicated that cryoconite holes on Exploradores glacier showed the highest richness and evenness. Samples on the Exploradores were collected close to the glacier terminus, surrounded by a rich evergreen broadleaf vegetation, and in an area with abundant supraglacial debris and frequented by tourists. The higher biodiversity of this large, low-altitude glacier, compared to that of the small, high-altitude Iver and East Iver glaciers is not surprising, as the rich evergreen broadleaf forest that surrounds the tongue of the first glacier can be the source of a richer and more diverse bacterial community than the bare ground surrounding the other ones. However, it is more surprising that the alpha biodiversity of the large, low-altitude Perito Moreno was intermediate and similar to that of the Morado glacier. Interestingly, Perito Moreno was the southernmost glacier among those we collected, and was surrounded by a less diverse forest, dominated by southern beeches, *Nothofagus* ssp. than that of Exploradores, while Morado was the glacier where samples were collected at the lowest altitude among the three glaciers near Santiago. We may therefore speculate that a broad gradient related to altitude and general climate conditions of the area surrounding the glacier may somehow affect its biodiversity. For instance, among the most abundant orders, Cytophagales were more abundant on high than on low-elevation glaciers (Fig. 5b). A similar pattern was observed for the Micrococcales and Chitinophagales (Fig. 5c–k) with the only exception of Iver.

In summary, we provide the first-ever description of the bacterial communities of cryoconite holes of glaciers in South America, specifically in the Southern Andes. This study thus fills an important gap of knowledge as almost no information was previously available on the cryoconite holes of this continent, and opens the possibility of future biogeography analyses including samples from almost every important glacial area of the world. The five glaciers we investigated are still a too small sample for thoroughly assessing the ecological processes that control cryoconite hole bacterial communities, and a larger set of environmental variables should also be considered, but we hope this study can be the basis for further investigations aiming at a deeper understanding of these extreme environments.

## Methods

### Study areas

Morado Glacier is a valley glacier located about 60 km South-East of Santiago (Chile). It covers 1.1 km^2^ and its altitudinal range is between 3535 and 4604 m a.s.l. It is a small glacier, highly crevassed and partly debris-covered in the ablation area, terminating in a small lake. Iver Glacier is a small mountain glacier located on Cerro El Plomo about 30 km North-West of Santiago (Chile). It has a surface area of 1.64 km^2^ and its altitudinal range is between 4296 and 5358 m a.s.l. It is a steep glacier, partly debris covered in the ablation area. East Iver is a small mountain glacier with a surface of 0.3 km^2^ located a few hundred meters to the East of the Iver Glacier at Cerro El Plomo. Exploradores Glacier is a valley glacier located in Chilean Patagonia. It covers 85 km^2^, it is 19 km long and its altitudinal range is between 158 and 3735 m a.s.l. The frontal area of the ablation zone is debris-covered. Perito Moreno Glacier is a valley glacier located in the Argentinian Patagonia. It covers 263 km^2^, it is > 32 km long and its altitudinal range is between 190 and 2800 m a.s.l. The glacier snout ends in a lake, losing most of its mass through calving processes. Supplementary Material Table S1 reports the coordinates of each sampling location.

### Sample collection

Cryoconite samples were aseptically collected in falcon tubes in 2017 and 2018 with a spoon sterilized with alcohol before collecting each sample (Table 2) from the ablation zone of each glacier, in debris-free areas close to the glacier margin (Perito Moreno) or the glacier terminus (all the other glaciers, see Fig. 1). We collected samples from 15 randomly chosen cryoconite holes in an accessible area of < 500 m on each glacier, avoiding the holes where a water flow was present. At each hole, we also recorded dissolved O_2_ concentration and pH with a portable oximeter/pH meter (HACH LANGE HQ40D, Loveland, CO, USA). Samples were kept at about 0 °C using glacier ice for 1–3 days while in the field and then stored at − 20 °C until DNA extraction. Preliminary studies indeed showed that when samples cannot be immediately frozen e.g. because collection occurred in remote areas, this is the storage strategy that alters least the bacterial communities^51^.

**Table 2:** Date of sampling of each glacier, coordinates of the approximate centre of the sampling area and (local) time of sampling of the first and last cryoconite hole.

Glacier	Date	Latitude	Longitude	Local time
Morado	18/02/2018	33.74661 S	70.05877 W	17:32–18:34
Iver	21/02/2018	33.25443 S	70.22703 W	12:32–15:05
East Iver	22/02/2018	33.25679 S	70.21540 W	13:11–14:46
Exploradores	01/03/2018	46.51843 S	70.17678 W	12:36–15:15
Perito Moreno	29–30/03/2017	50.51450 S	73.12306 W	10:52–17:48

### DNA extraction and sequencing

DNA was extracted from 0.7 g of cryoconite with the FastDNA Spin for Soil kit (MP Biomedicals, Solon, OH, USA) according to the manufacturer’s instructions. DNA sequencing was performed on the V5-V6 hypervariable region of the 16S rRNA gene with the 783F (CAGGATTAGATACCC) and 1027R (CGACRRCCATGCANCACCT) primers as previously described in Pittino et al.^10^. Sequences were then demultiplexed according to the indexes and clustered in Amplicon Sequences Variants (ASVs) with DADA2^28^. ASVs were then taxonomically classified using the SILVA classifier^52^ keeping the full classification only of the taxa attributed with a confidence of 0.8 or higher^53,54^.

### Statistical analyses

Variation among glaciers in pH and [O_2_] was investigated through ANOVA followed by Tukey post-hoc tests.

Singletons (ASVs present once in one sample only) were removed from the analyses because they can inflate the variance explained by multivariate tests^55^. Alpha-diversity was measured using the Shannon diversity index, which accounts for both the richness and the evenness of the species^56^, and the Gini inequality index, which is an index of inhomogeneity largely used in economics^57^. Gini index ranges from 0 to 1 and low values indicate a homogeneous distribution of the species in the community, while values close to one indicate a heterogeneous distribution^57^. These indices were calculated on a dataset rarefied to 7500 sequences per sample, i.e. slightly less than the minimum number of sequences in a sample (see results). Samples with < 7500 sequences (N = 6, all from Morado Glacier) were discarded from alpha diversity analyses.

Beta diversity analyses were conducted on a non-rarefied dataset, and were based on the Hellinger distance, which depends on the differences in the ASV proportion among samples, decreases the importance of ASV abundance over occurrence and avoids the double-zero problem when comparing ASV composition among samples^58^. We performed a redundancy analysis (RDA) to quantify the variation of community structures among glaciers (five-level factor), and according to dissolved oxygen concentration and pH of the water above the sediment. The last two variables were mean-centred within glacier before the analyses (i.e. we subtracted from the values recorded at each glacier their mean value). Centred variables were called ΔpH and Δ[O_2_], respectively. Post-hoc tests (Tukey method) were also performed to assess pairwise differences in the structure of bacterial communities between glaciers, correcting P-values for multiple testing according to the false discovery rate (FDR) procedure^59^.

Generalized least-square (GLS) models accounting for the heterogeneity of variance among glaciers were also used to investigate changes in alpha diversity indices according to ΔpH, Δ[O_2_], and glacier (five-level factor). Variation in the abundance of the nine most abundant orders (see below) according to the variables that significantly affected the structure of bacterial communities identified by the RDAs was investigated by generalized linear models (GLMs) assuming a Poisson distribution and correcting for overdispersion. In these models, the dependent variable was the non-rarefied number of sequences assigned to that order per sample. We also included the log-transformed total number of sequences per sample as an offset. With this structure, the Poisson GLMs model the relative abundance of each bacterial order according to the predictors. Also in these cases, P-values were corrected using the same FDR procedure as above. Analyses were performed with R 3.5.1^60^ with the *vegan*, *BiodiversityR*, *multtest*, and *multcomp* packages.

## Supplementary Information

Supplementary Information.

## Data Availability

Sequences were submitted to NCBI (http://www.ncbi.nlm.nih.gov/bioproject/745072).
