# Supplementary material for: Geographical variability of bacterial communities of cryoconite holes of Andean glaciers
Source: Sci Rep. 2023 Feb 14;13:2633. doi: 10.1038/s41598-022-24373-5 (PMC9929092; doi:10.1038/s41598-022-24373-5)
Supplement: Supplementary file 1 — Supplementary Information. [file 41598_2022_24373_MOESM1_ESM.pdf]

Supplementary Materials

Geographical variability of bacterial communities of cryoconite holes of Andean glaciers

F. Pittino, R. Ambrosini*, M. Seeger, R. S. Azzoni, G. Diolaiuti, P. Alviz Gazitua, A. Franzetti

* roberto.ambrosini@unimi.it

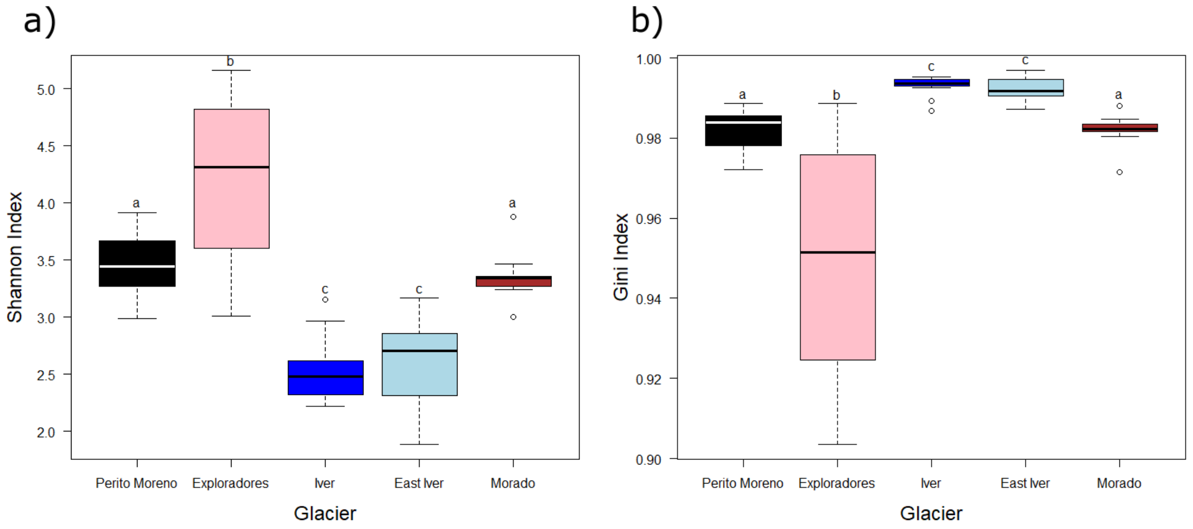

Figure S1 Boxplots of Shannon (a) and Gini (b) diversity indices of cryoconite hole bacterial communities. The thick lines represent the median, boxes upper and lower limits the 25th and the 75th percentiles respectively, whiskers the 5th and the 95th percentiles respectively, dots represent the outliers, and different letters indicate significant differences at post-hoc tests.

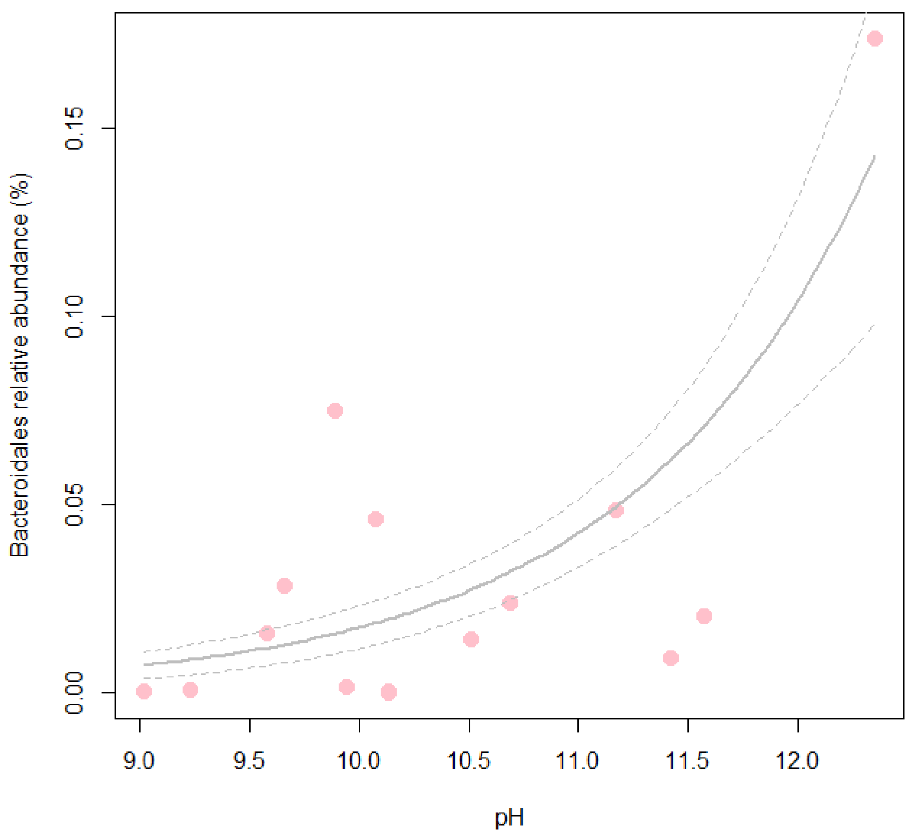

Figure S2 Trend of Bacteroidales on the Exploradores glacier according to pH. The thick continuous line represents the trend fitted according to the coefficient of the Poisson GLM and the dashed lines its standard error.

Table S1. Coordinates of the sampled cryoconite holes.

Sample Name	Longitude	Latitude	Glacier
GSA.PT1.29	-73.12267	-50.51415	Perito Moreno
GSA.PT1.30	-73.12267	-50.51415	Perito Moreno
GSA.PT2	-73.12238	-50.51408	Perito Moreno
GSA.PT3	-73.12292	-50.51413	Perito Moreno
GSA.PT4	-73.12468	-50.51635	Perito Moreno
GSA.PT5	-73.12343	-50.51525	Perito Moreno
GSA.PT6	-73.12395	-50.51385	Perito Moreno
GSA.PT7	-73.12310	-50.51393	Perito Moreno
GSA.PT8	-73.12310	-50.51390	Perito Moreno
GSA.PT09	-73.12347	-50.51352	Perito Moreno
GSA.PT10	-73.12228	-50.51342	Perito Moreno
GSA.PT11	-73.12290	-50.51423	Perito Moreno
GSA.PT13	-73.12277	-50.51525	Perito Moreno
GSA.PT14	-73.12298	-50.51522	Perito Moreno
GSA.PT15	-73.12290	-50.51483	Perito Moreno
GSA.E1	-70.17508	-46.51872	Exploradores
GSA.E2	-70.17508	-46.51878	Exploradores
GSA.E3	-70.17514	-46.51931	Exploradores
GSA.E4	-70.17542	-46.51919	Exploradores
GSA.E5	-70.17661	-46.51836	Exploradores
GSA.E6	-70.17667	-46.51847	Exploradores
GSA.E7	-70.17664	-46.51847	Exploradores
GSA.E8	-70.17703	-46.51814	Exploradores
GSA.E9	-70.17706	-46.51817	Exploradores
GSA.E10	-70.17742	-46.51814	Exploradores
GSA.E11	-70.17753	-46.51817	Exploradores
GSA.E12	-70.17753	-46.51816	Exploradores
GSA.E13	-70.17797	-46.51822	Exploradores
GSA.E14	-70.17819	-46.51817	Exploradores
GSA.E15	-70.17828	-46.51792	Exploradores
GSA.IV1	-70.22780	-33.25550	Iver
GSA.IV2	-70.22778	-33.25550	Iver
GSA.IV3	-70.22780	-33.25470	Iver
GSA.IV4	-70.22780	-33.26980	Iver
GSA.IV5	-70.22780	-33.25470	Iver
GSA.IV6	-70.22770	-33.25470	Iver
GSA.IV7	-70.22770	-33.25460	Iver
GSA.IV8	-70.22770	-33.25460	Iver
GSA.IV9	-70.22780	-33.25410	Iver
GSA.IV10	-70.22780	-33.25400	Iver
GSA.IV11	-70.22680	-33.25210	Iver
GSA.IV12	-70.22640	-33.25180	Iver
GSA.IV13	-70.22560	-33.25140	Iver
GSA.IV14	-70.22560	-33.25139	Iver
GSA.IV16	-70.22559	-33.25139	Iver

Table S1. Continued.

Sample Name	Longitude	Latitude	Glacier
GSA.IVE1	-70.21470	-33.25640	East Iver
GSA.IVE2	-70.21470	-33.25680	East Iver
GSA.IVE3	-70.21472	-33.25680	East Iver
GSA.IVE4	-70.21480	-33.25690	East Iver
GSA.IVE5	-70.23150	-33.25690	East Iver
GSA.IVE6	-70.23148	-33.25689	East Iver
GSA.IVE7	-70.21530	-33.25680	East Iver
GSA.IVE8	-70.21530	-33.25680	East Iver
GSA.IVE9	-70.21530	-33.25680	East Iver
GSA.IVE10	-70.21530	-33.25680	East Iver
GSA.IVE11	-70.21530	-33.25681	East Iver
GSA.IVE12	-70.21540	-33.25680	East Iver
GSA.IVE13	-70.21541	-33.25680	East Iver
GSA.IVE14	-70.21540	-33.25690	East Iver
GSA.IVE15	-70.21540	-33.25690	East Iver
GSA.M1	-70.05863	-33.74703	Morado
GSA.M2	-70.05828	-33.74697	Morado
GSA.M3	-70.05837	-33.74646	Morado
GSA.M4	-70.05840	-33.74673	Morado
GSA.M5	-70.05844	-33.74710	Morado
GSA.M6	-70.05845	-33.74664	Morado
GSA.M7	-70.05861	-33.74685	Morado
GSA.M8	-70.05888	-33.74703	Morado
GSA.M9	-70.05783	-33.74506	Morado
GSA.M10	-70.05878	-33.74612	Morado
GSA.M11	-70.05886	-33.74721	Morado
GSA.M12	-70.05886	-33.74721	Morado
GSA.M13	-70.05871	-33.74751	Morado
GSA.M14	-70.06023	-33.74558	Morado
GSA.M15	-70.06023	-33.74558	Morado
